# Analysis of the quality of seasonal malaria chemoprevention provided by community health Workers in Boulsa health district, Burkina Faso

**DOI:** 10.1186/s12913-019-4299-3

**Published:** 2019-07-10

**Authors:** Gountante Kombate, Georges Guiella, Banza Baya, Luc Serme, Alice Bila, Slim Haddad, Abel Bicaba

**Affiliations:** 1 Societé d’Études et de Recherche en Santé Publique, Ouagadougou, Burkina Faso; 2 Institut Supérieur des Sciences de la Population, Université de Ouagadougou, Ouagadougou, Burkina Faso; 3 Faculté de Médecine, Université Laval, Quebec City, Canada

**Keywords:** Care quality, Community health workers, Seasonal malaria chemoprevention, Boulsa, Burkina Faso

## Abstract

**Background:**

Since 2014, the Burkina Faso government has made Seasonal Malaria Chemoprevention (SMC) a priority in its strategic plan to fight against malaria among children aged from 3 to 59 months. Very few studies have examined the care provided by community health workers in the framework of this strategy. The purpose of this study was to evaluate the level of quality of care provided by the latter.

**Methods:**

This was a mixed study. The quantitative component consisted of a non-participant observation of community health workers during the administration of care. The qualitative component consisted of one-on-one interviews with community health workers, child caregivers and head nurses. Five dimensions (organizational accessibility, interpersonal relationship, technical competence, safety of care and satisfaction of child caregivers) adapted from the Donabedian quality of care model were used to assess the quality level of care. The Corlien et al. Health Systems Research Program Implementation Scale was used to establish quality scores for each of the five dimensions. The study sites were the health centers located in the administrative centers of the 4 communes of the health district of Boulsa. The data were collected during the first cycle of the 2017 SMC campaign.

**Results:**

A total of 14 active pairs (28 CHWs) were observed and 40 in-depth interviews with community health workers, Head nurses in duty and community leaders were conducted. The results show that community health workers worked in pairs. They had all received SMC training and possessed equipment to do their job. The dimensions of organizational accessibility and satisfaction of the caregivers were rated as good. The dimensions of interpersonal relationship and technical competence were judged to be of an acceptable score. Safety of care was judged to be of a low-level score. The overall quality of care was considered acceptable.

**Conclusion:**

The results of this study have shown that despite the difficulties faced by community health workers, they manage to deliver acceptable quality of care. Their use would be an asset for SMC in particular and for the health system in general.

**Electronic supplementary material:**

The online version of this article (10.1186/s12913-019-4299-3) contains supplementary material, which is available to authorized users.

## Background

In March 2012, the World Health Organization (WHO) recommended a new strategy called Seasonal Malaria ChemoPrevention (SMC), to prevent the peak of malaria-related morbidity and mortality among children aged from 3 to 59 months. It was recommended particularly for countries of the Sahel sub-region, where malaria is endemic [1]. SMC consists of intermittent administration of a complete Sulfadoxine-Pyrimethamine (SP) and Amodiaquine (AQ) treatment during the high-transmission season. The goal is to maintain therapeutic levels of these drugs in the blood during the period when the risk of contracting malaria is the highest [1, 2]. In view of the evolution of the malaria epidemic in the countries of sub-Saharan Africa, this strategy was adopted and implemented in several countries, such as Burkina Faso in 2014 [2–5]. In this country, malaria is highly transmissible during the months of July through October [4]. During this period, it is highly recommended to administer the treatments [6]. The strategy is to administer three doses of medication per month, of which the first dose is given or supervised by a community health worker (CHW) or volunteer in the community. The last two doses are given to the child caregiver with instructions on following up with the treatment 2 days after the initial supervised dose [1]. In the SMC’s framework strategy, all community-level agents (community health workers and some volunteers as needed) involved in drug delivery are referred to as Community Distributors (CD) [7]. These community health workers have previously participated in many other health programs at the community level since the launch of primary health care at the Alma Atta conference in 1978 [8]. Several studies have demonstrated the importance of CHWs in strengthening health system in many African countries [9, 10]. On the other hand, some authors do not share the same opinion [11, 12]. They argue that most of the studies on the quality of care provided by CHWs specifically in the context of home-based malaria management were evaluated under controlled implementation conditions (pilot project or randomized trials). The quality of care provided by CHWs on national implemented program under routine conditions has been poorly studied [13, 14]. The fact is, since the implementation of SMC throughout Africa, very few studies have looked at the quality of care provided by CHW. For example, in Burkina Faso, no study has been done until now. Studies that have addressed the quality of care of CHWs have been done as part of malaria control interventions other than SMC. Therefore, this study was conducted to assess the quality of SMC-led care in the Boulsa health district in Burkina Faso. Specifically, it is consisted in the assessment of the quality of SMC provided by CHWs based on five dimensions adapted from the quality of care model of Donabedian [15, 16], and to identify certain factors that can influence this quality of care.

## Methods

### Conceptual framework

To assess the level of quality of care for CHW in the context of SMC, we used the conceptual framework described by Donabedian [15, 16]. According to Donabedian, three determinants (structures, processes, results) make it possible to assess the quality of care. The choice of Donabedian’s model can be explained by its wide use and by the fact that it allows the conceptualization of mechanisms underlying the quality of care of CHW [17].

### Context, study design and sampling

This study was conducted in the Boulsa health district, located in the north-central region of Burkina Faso. It includes four municipalities, only one of which is urban. According to the statistical yearbook of the Ministry of Health in 2016, the total population of the district is estimated at 209,986 inhabitants, of which 19.22% are children under five and with an average density of 64.5 inhabitants per km^2^. Malaria is the leading cause of morbidity and mortality with 62.21% of cases in the top ten pathologies prevalent in the district of Boulsa in 2016 [18].

A mixed methods research design was used. The participants in this study were CHWs, child’s caregivers, head nurses and community leaders. Purposive sampling was used to select the different participants [19]. The principle was to include the various actors involved in the SMC and belonging to public Health and Social Promotion Centers (HSPC) located in the capitals of each of the four municipalities that organized the SMC in 2017. This technique was chosen considering some aspects such as geographical accessibility and the population density of the study sites, which allowed diversity in information from the participants [20].

Three tools were used for the data collection including semi-structured interview grids for the Head Nurses and CHWs (Additional files 1 and 2), which provided insights into the various SMC medication administration procedures, working conditions and challenges; a non-participant observation grid that was used to collect data directly related to the practices of CHWs in households. These observations focused on the first four quality of care dimensions: organizational accessibility, interpersonal relationship, technical competence and care security. Since CHWs worked in pairs, we made ten observations per pair and per day. One observation was the entire process of administering SMC drugs to a child. As a repository of the type and nature of the information to be collected, an observation guide in the form of a checklist was developed based on the World Health Organization SMC’s handbook [8]. A questionnaire addressed to the child caregivers in the SMC’s framework evaluates the fifth dimension of quality of care and the satisfaction of the caregivers (Additional file 3).

The interviews were conducted to the point at which no new information was elicited [21]. The semi-structured interview grids were adapted to each category of respondents. The questionnaire and interviews were administered in French or in the local language “Moore”, depending on the respondent’s preference.

### Data collection

Data were collected in July 2017. This period was the first time that CHWs were requested for the 2017 SMC campaign and at the beginning of the high malaria transmission season in Burkina Faso, which extends from July to October.

The collect is carried out by four research assistants of the society of studies and research in public health (SSRPH) whom understand and speak correctly the local language “Moore”. Participants were informed that SSRPH is located in Ouagadougou and is independent of the health system. The interviews lasted approximately 1 h and were collected using a dictaphone and a parallel note taking. The transcripts of the interviews were done systematically in French at the end of each day. The data was checked and validated daily.

### Data analysis

Direct content analysis using a deductive approach was the chosen method [22, 23]. This approach allows analysis of representations and practices in qualitative studies [24]. The theoretical framework of Donabedian (1992) [16] was used to organize the initial coding. This initial coding was refined by applying it to the first transcripts and adding subcategories and combination codes. Similar codes have been grouped into categories. The analysis was carried out using the qualitative software Nvivo (version 10).

For the quantitative part, the observations data were entered into an Excel file and analysed with the SPSS version 20 software. The quality of care assessment approach based on the Averting Maternal Death and Disability [25] from the Columbia University School of Public Health was used for the quantification of each of the five quality dimensions. According to this approach, it is necessary to identify and sum up the positive and negative responses obtained for each dimension (a positive response is a well-executed task and a negative response, a bad or not performed task). Subsequently, we divided the number of positive responses by the sum of the positive and negative responses. The result multiplied by 100 gives the percentage of positive responses for each quality of care dimension. In order to evaluate the results obtained from each of the five dimensions, the scale of evaluation and implementation of the health systems research programs of Corlien et al. [26] was used. For each dimension, we assigned scores ranging from 1 to 3. A percentage of positive responses ≥80% receives a score of 3 and corresponds to a good level of quality of care. A percentage of positive responses < 80% but ≥60% receives a score of 2 and corresponds to an acceptable level of quality of care. Finally, a percentage of positive responses < 60% receives a score of 1, which corresponds to a low level of quality of care.

## Results

Overall twenty-eight (28) CHWs divided into 14 teams of two were observed for a total of 140 non-participant observations. A total of thirty-four (34) in-depth interviews were conducted (28 CHWs, four HNs, and two LCs).

### Sociodemographic characteristics of CHWs

CHWs (*n* = 28) were predominantly men (74%) with an average age of 43 years (SD =14.6). They had an average of 5.14 years (SD = 4.72) of work experience as a community health worker (CHW). In the context of SMC as community distributors, they had on average 1.53 years (SD = 0.92) of experience. Most of them (57%) were farmers, and 69% of them had a primary level of education and more. All (100%) were trained on SMC before the start of the campaign.

### Characteristics of children who received SMC by age and sex

Out of the children who received SMC medication (*n* = 140), 65% were children aged 12 to 59 months, compared with 35% of children aged 3 to 11 months. They were predominantly male (57.5%).

### Sociodemographic characteristics of child caregivers

A total of the 33 child caregivers responded to the questionnaires, 54% were the mothers of the children, 10% were co-wives, 27% were grandmothers. The average age was 36 years (SD = 15.27). 88% were women, and 69% of them had no level of education (Additional file 4).

Table 1 presents a summary of the results dimension by dimension.

**Table 1:** Summary of dimensions of assessment of care quality

Quality criterion	Positive answers	Negative answers	Total answers	% of positive responses	Score
Organization of CDs’ work					
There are two CDs per team	140	0	140	100	3
CDs are both trained	140	0	140	100	3
Tasks are distributed	110	30	140	100	2
Availability of care equipment	140	0	140	100	3
Availability of drugs	140	0	140	100	3
All target children have access to care sssstraitement	140	0	140	100	3
All tools are used correctly	96	44	140	69	2
Total	906	74	92	92	19
Interpersonal relationship between CD and child caregivers					
Greetings from the caregivers	117	23	140	83	3
Using a simple vocabulary	110	30	140	78	2
No conflict situations	140	0	140	100	3
Total	574	126	700	82	12
Technical skills of CDs					
CDs are looking for all children under 5 years	136	4	140	97	3
They ask the age of the children	137	3	140	98	3
They are looking for allergic reactions	43	97	140	31	1
They ask if the child has already received SMC	44	96	140	31	1
They administer effectively and gently	106	34	140	76	2
They respect the doses of the drugs	120	20	140	86	3
They provide and give information about taking the 2nd and 3rd doses	140	0	140	100	3
They refer to ineligible children (sick)	106	43	140	76	2
They fill the tools correctly	109	33	140	78	2
They talk about possible allergic reactions	41	99	140	29	1
They do the marking of the Households	107	33	140	76	2
Total	1089	451	1540	71	23
Care safety for children					
CDs are dressed correctly	86	54	140	61	2
Cleanliness of the place of administration	107	33	140	76	2
They wash their hands with soap	60	80	140	43	1
They use clean water	110	30	140	78	2
They use clean equipment	80	60	140	57	1
They dilute the drugs correctly	110	30	140	79	2
The material is washed after each treatment	28	112	140	20	1
They observe 30 min after the administration	16	124	140	11	1
Total	597	523	1120	53	12

#### Assessment of quality of care according to organizational accessibility

Seven criteria made it possible to assess this dimension (Table 1). Each criterion received a rating of 3, and the total score was 19/21 (90%) which reflects a good organizational level. Most CHWs were married men who completed primary school or more. They were already CHWs with long experience before being CHWs in the SMC. They were in pairs and worked in their own community. As for the Head Nurses on duty, they noted the need to train CHWs and put them in pairs properly. They considered to put at least one member on the team who can read and write, because of the filling tools that were in French (child administration card and reference sheet).

*“We are two here, there is one who administers the treatment to the children, and there is one who keeps a card, and then he marks there, to facilitate the tasks. But we work together. If someone forgets something the other can do it. Ha!! It will be difficult; if it’s one person, it will be too difficult”.* (CHW 17)

#### Assessment of the quality of care according to the interpersonal relationship

Five criteria made it possible to assess this dimension (Table 1). The total score was 12/15 (80%) which reflects a good interpersonal relationship. Indeed, the results of the observation reveal that the observed CHWs showed respect towards the caregivers. They used simple and clear language. They ensured that child caregivers understand and used work tools appropriately. But they did not give enough time to the child caregivers to express themselves in return. In our interviews about attitudes towards child caregivers, the CHWs described very well the attitude to be adopted to be well received and pass the information in households.

*“Ah! When we go back there [in a concession], we greet the parents of the children first, after we explain them why we are there and what medicine we want to give to children. If the parents there accept, they will bring the children we will give them medicine”.* (CHW 18)

The explanation of care at each stage of medication administration and listening to the child caregivers in return are the only points that are lacking in this dimension. After obtaining the agreement of the child caregivers, some CHWs did not take enough time to administer the drugs. They said they had a lot to do and was quick to move on to another household. Additional details and details in the information provided would enable child caregivers to have a better understanding of SMC.

#### Assessment of the quality of care according to technical competence

Of the 11 criteria used to assess this dimension (Table 1), the total score was 23/35 (70%), which reflects the technical competence of the CHWs at an acceptable level. For all the CHWs observed, most of the criteria were respected. Some CHWs have encountered difficulties related to the eligibility of the child, especially if the child has or has not previously received SMC, checking for an allergic reaction to medications, and sometimes to determine the exact age of the child. There was also poor filling of some tools and a lack of precision regarding information on adverse effects that may occur after administration of the drug. During the interviews, the CHWs relied on attitudes and practices to describe the essential tasks to be provided and the techniques. To the question of how CHWs determine the ages of children, whether they have been reported to the registry office or not, they describe the following processes:

*“Well, we ask mum first to know the age of the child. But if she does not know it, one asks at the same time the vaccination card of the child, or if the child has health record also, it is good. But sometimes the child is also asked to take his left hand to touch his right ear through the top of his head; if his left hand touches the ear, the child does not take. But if it does not touch it, it means that he is not yet 5 years old, we can give him now”.* (CHW 22)

Regarding the shortcomings observed in this dimension, the CHWs said they had a lot to do and did not have time to respect everything. Some of them even acknowledged that sometimes certain gestures or information to give them escaped and that they ended up forgetting them in the routine of the activities.

#### Assessment of the quality dimension according to care safety

Eight criteria allowed the appreciation of this dimension (Table 1). The total score was 12/24 (50%) which reflects a low level of safe care. The observed CHWs had trouble complying with some of the rules of washing hands and equipment with soap and water. Considering the different stages of safe care, the fact that sanitary rules where not respected was linked mostly to the lack of sanitary materials to guarantee safety when providing care, as the following extract illustrates it:

*“We have not been given even soap, what you see there, it is with our own money that we managed to buy it. Well, that’s why..., we cannot properly apply the techniques that Commandant [Head Nurse] showed us at HSPC (the Health and Social Promotion Center) when we were having training there”.* (CHW 18)

#### Assessment of quality according to the satisfaction dimension of the managers

For this dimension, a questionnaire was submitted to 33 caregivers. The percentage of positive responses was 267/330 or 81%. And according to the scale of Corlien et al. (1993), it corresponds to a good level of satisfaction of the caregivers. Most child caregivers surveyed reported being satisfied with the work of CHWs and the attitude of respect they had when they visited households. Compared to the knowledge about SMC and the benefits to help mobilize the population, the community leaders interviewed gave their point of view:

*“Yes, we know that it is to fight against malaria for children. It works well eh, since it came to our village here, children do not get sick like that. That’s why every time when it comes that way, the Commandant [the head nurse] comes to tell me that we will tell the people of the village that it started. They will stay with the children at home so that the CHWs there will come and give them drugs”.* (CL 1)

#### Overall quality of care assessment of SMC

Table 2 shows the overall assessment of SMC quality of care. The arithmetic sum of the five dimensions is 78%, which reflects an overall quality of SMC care of acceptable level. Nevertheless, it must be recognized that difficulties hindered the smooth running of CHWs. This is the example of weather (Wintering period) and the number of children to be administered per day:

*“Yes, that’s what I said; we do not have much time, we have to do 50 children a day. And then you can get up in the morning at 6 o’clock and then there is rain too, which is there, the time also ahead. So, oh when we start there, we cannot spend all the time at one place like that. Well, sometimes we cannot take our time to do the job well”* (CHW 10).

**Table 2:** Overall Quality of Care Assessment of SMC

Dimensions of care quality	Expected maximum Scores	Obtained maximum Scores	Ratio obtained Score on expected score
Organizational accessibility	21	19	0.90
Interpersonal Relationship	15	12	0.80
Technical Skills	33	23	0.70
Care Security	24	12	0.50
Satisfaction of leaders	330	267	0.81

## Discussion

The main objective of this study was to assess the SMC’s quality level provided by CHWs in the Boulsa health district in Burkina Faso. We found that the quality of care provided by CHWs during SMC is acceptable despite the difficulties encountered. When looking more specifically at the dimensions of quality of care in this study, the results are variable.

### The organizational accessibility dimension

CHWs enrolled as part of the study were all trained prior to the start of the SMC campaign and well organized around the job. The WHO (2004) [27] emphasizes that the training of all providers (formal health workers as well as community workers) is an essential component to improve disease prevention and management. Among our CHWs 31% had no education, but they were still solicited because of their training and many years of experience as CHW. Regardless of their literacy level, all CHWs received the same training and had the same amount of responsibility on the field. Both the illiterate and literate CHWs had comparable performances and operational inputs. As reported in other studies, CHWs expressed the need for bicycle to reduce fatigue from walking, and offer quality care to the community. Indeed, cycling can reduce travel times between concessions, especially in rural areas where they are far from one another [11, 28].

### The interpersonal relationship dimensions

The criterion not well observed by the CHWs was the attentive listening of the caregivers. They did not ask for their consent. They attributed this shortfall to a lack of time related to the excessive workload. However, obtaining collaboration and listening carefully the patient is a very important component of service quality. In Senegal, Faye in showed that CHWs involved in the preventive treatment of seasonal malaria did not take enough time to obtain the individual consent of the child’s caregiver in the concessions [29]. It is rather the consents obtained from the community leaders that they took into consideration. However, the consent of leaders, regardless of its form, does not dispense with individual consent of participants [30]. Also, the fact that the CHWs come from the locality where they work leads them not to perceive the need to ask permission from the parents [29].

### The technical competence dimensions

All CHWs administered the first dose of treatment themselves. 76% of sick children were referred, and all the caregivers received information on the remaining doses they themselves administered at home the other days. The CHWs demonstrated a mastery of SMC’s administration protocol. The study conducted by Smith et al. on the performance of CHWs in Madagascar on the management of childhood illnesses, found similar results [31]. This study proved that the skill level of CHWs was acceptable with an average performance of 75%. Nzayirambaho et al. in Rwanda reported similar results [19].

In our study, lack of supervision was reported by ASC as a significant problem. They say that this should remind them of some important aspects when administering. Some studies in Rwanda [12] and Kenya [32] have shown that CHWs perform basic care activities at the community level, but also have shortcomings in the quality of care they provide. Supervision could therefore help to correct the inadequacies of CHWs and maximize their effort [11, 33]. However, the finding is that CHWs supervision often lacks rigor in community health interventions in developing countries [34].

### The security dimension of care

The level of care security was rated low, revealing a deficiency. Systematic washing of hands and equipment with soap and water before and after medication was not followed in all cases. CHWs had the reflex to wash their hands in only 42.5% of cases and the material in 20% of cases. A study of community-based management of malaria’s cases by CHWs in the Saraya Health District in Senegal found similar results. Of the 9 criteria for quality of care measurement in rapid diagnostic tests (RDTs), hand washing and wearing gloves were not performed by any of the 30 CHWs evaluated [31]. Once again, lack of time and equipment were the reasons reported by CHWs. The CHWs linked this deficit to an important workload [29]. In our study, CHWs should administer medications in 80 households a day. The safety of care situation noted in our study is very worrying and should attract the attention of the health authorities especially in a context marked by the omnipresence of transmitted diseases.

### The satisfaction dimension of the child caregivers

The level of satisfaction of child caregivers with the work done by CHWs is rated as good (81%). Indeed, child caregivers respect and value the work of CHWs. They recognize that this is a difficult work and that they give the best of themselves. Our results are consistent with those of USAID study conducted in Madagascar in 2013 on community-based management of malaria. In this study, community satisfaction was 76% [35]. The results of the Nzayirambaho et al. study in Rwanda also showed a 100% satisfaction of the child caregivers interviewed about the work done by CHWs [19]. This is probably an additional source of motivation for CHWs.

### Limits of the study

This study is not without limits. First, it is the number of CHWs enrolled in this study. This is related to the complexity of the door-to-door drug administration strategy and the geographic accessibility of the study area. We would then calculate a sample that is statistically representable giving a larger number of CHWs. This would certainly have identified much more diversity in the quality of SMC care provided by CHWs in the Boulsa Health District. Second, the observational technique used also has limitations. CHWs were able to provide better care than normal because they were observed. A phenomenon often called “Hawthorne effect” [36], which would also have introduced a positive bias in relation to the quality of care normally received by children. Nonetheless, using the SMC’s administration guide proposed by WHO as the standard for CHW’s quality assessment was the strength of this study. This guide allowed us to provide valuable results on the quality of SMC care received by children from 3 to 59 in the Boulsa health district. Another strength of this study was the assessment of the quality of care provided by the CHWs, which was done in the communities where they work, unlike other studies based on health facilities. This allowed us to witness a “real” evaluation of community health policy.

## Conclusion

CHWs manage to deliver acceptable quality care despite their volunteer status and the challenges they faced. There are still shortcomings in terms of hygiene and obtaining the consent of caregivers and some techniques in the process of administration of medicines. Ongoing training in SMC, monitoring and supervision of CHWs in the field are of paramount importance to maintaining the quality of care in this program. Their use remains an asset for SMC and for the health system in general.1,2,3,4

## Additional files

Additional file 1: Interview grid for the Head Nurses. (PDF 204 kb)

Additional file 2: Interview grid for community health workers (CHWs). (PDF 287 kb)

Additional file 3: Questionnaire_ Satisfaction of child caregivers. (PDF 125 kb)

Additional file 4: Questionnaire_Socio-demographic characteristics of CHWs. (PDF 120 kb)

## Data Availability

The data analyzed for this manuscript are available from the corresponding author on reasonable request.

## Abbreviations

AQ: Amodiaquine
CD: Community distributors
CHW: Community health workers
CL: Community leader
HN: Head Nurse
HSPC: Health and Social Promotion Centers
RDT: Rapid diagnostic tests
SMC: Seasonal Malaria chemoprevention
SP: Sulfadoxine-pyrimethamine
SSRPH: Society of Studies and Research in Public Health
