# Supplementary material for: Analysis of the quality of seasonal malaria chemoprevention provided by community health Workers in Boulsa health district, Burkina Faso
Source: BMC Health Serv Res. 2019 Jul 10;19:472. doi: 10.1186/s12913-019-4299-3 (PMC6617895; doi:10.1186/s12913-019-4299-3)
Supplement: Supplementary file 1 — Interview grid for the Head Nurses. (PDF 204 kb) [file 12913_2019_4299_MOESM1_ESM.pdf]

Interview grid for the Head Nurses

Name ……………………………………… ID…………………. Age …………

District…………………………………… Health centre ……………………………..

Date of interview…………………………………….. Interview conducted by……………

Theme1: Role and recruitment of CHWs

- ✓ Could you talk to me about CHWs in your health area? How many CHWs are there for this programme?
- ✓ What are the criteria?
- ✓ Tell me what their role consist in in the SMC?
- ✓ Do you have newly-recruited CHWs for this year?

Theme2: HCDs’ training

- ✓ Have you given a training session to the CHWs for this SMC programme?
- ✓ What do you think about this CHWs’ training?
- ✓ How long did you hold this training?
- ✓ Was this time sufficient to you?

Theme3: Relations with the community

- ✓ Do you have any idea about the relations existing between the CHWs and the community in your health area? What does the community think about the CHWs?
- ✓ Does the community trust in the CHWs when they come to households to administer medicines?

Theme4: Organisation of the work

- ✓ Could you talk to me about the organisation of the CHWs’ work?
- ✓ Do you think that their number enables you to do best the work?
- ✓ Do you have any idea about the duties of CHWs’?
- ✓ What do the CHWs find difficult or easy about the SMC?
- ✓ What aspects of healthcare quality are the most difficult?

Theme5: Supervision

- ✓ Do you go on a supervisory excursion during the SMC?
- ✓ Could you talk to me about your supervision over the CHWs?
- ✓ What are your goals when you do a supervision?
- ✓ What makes the supervision difficult?

Thank you for your collaboration!
