# Supplementary material for: Analysis of the quality of seasonal malaria chemoprevention provided by community health Workers in Boulsa health district, Burkina Faso
Source: BMC Health Serv Res. 2019 Jul 10;19:472. doi: 10.1186/s12913-019-4299-3 (PMC6617895; doi:10.1186/s12913-019-4299-3)
Supplement: Supplementary file 2 — Interview grid for community health workers (CHWs). (PDF 287 kb) [file 12913_2019_4299_MOESM2_ESM.pdf]

Interview grid for community health workers (CHWs)

Name……………………………………………… ID…………. Age……………..

District……………………… Health Centre………………… Village……………….…….

Date of Interview …………….. Interview conducted by……………………………...

Theme1: Availability of resources and type of service

- ✓ How many of you are involved in Seasonal Malaria Chemoprevention (SMC) in your distribution area?
- ✓ Does this number enable you to do best the work?
- ✓ Have you received any training or retraining in as part of SMC?
- ✓ Have you got the necessary equipment to implement healthcare for children?
- ✓ Have you always been available for the SMC activities?

Theme2: Organisation and service provision

- ✓ How do you get organised to work?
- ✓ Is not one person sufficient for performing the work?
- ✓ Do you use other means to make easy the work? (concerning the identification of target children, attitudes to have before children guardians, administration techniques, hygiene during medical care)
- ✓ What are the key information that you provide child caregivers?
- ✓ Do you often practise the transfer of children to the local health centre? If yes, in what conditions?
- ✓ What are the steps that you must follow during the administration of medicine?

Theme3: Supervision and difficulties experienced

- ✓ Do you receive supervisons? If yes, what happens during the supervision visit?
- ✓ In your opinion, what are the major difficulties that you come across while providing children with the SMC medical care? In your opinion, what are the major challenges you face in providing SMC care to children?
- ✓ How are you motivated for the service you render?
- ✓ What should be done, according to you in order to improve the SMC programme?
- ✓ What raises your interest for this work?

Thank you for your collaboration!
