# Supplementary material for: Analysis of the quality of seasonal malaria chemoprevention provided by community health Workers in Boulsa health district, Burkina Faso
Source: BMC Health Serv Res. 2019 Jul 10;19:472. doi: 10.1186/s12913-019-4299-3 (PMC6617895; doi:10.1186/s12913-019-4299-3)
Supplement: Supplementary file 3 — Questionnaire_ Satisfaction of child caregivers. (PDF 125 kb) [file 12913_2019_4299_MOESM3_ESM.pdf]

Questionnaire No. ……

Questionnaire_ Satisfaction of child caregivers

A1	Date	/_____/_____/______
A2	District	
A3	Health centre	
A4	Village	
A5	Child caregiver's name	
A6	Sex	(1) Male<br>(2) Female
A7	Age	/____________
A8	Schooling level	(1) Unschooled<br>(2) Literate(local language)<br>(3) Primary school<br>(4) Secondary school<br>(5) University
A9	Relationship to child	(1) Biological mother<br>(2) Father<br>(3) Co-wife<br>(4) Grand-mother<br>(5) Grand-father<br>(6) Daughter/ son<br>(7) Other (to specify)/____
A10	Inquirer's name	
A11	Have the CHWs come to your home?	(1) Yes<br>(2) No
A12	Has the child got medicine from the SMC?	(1) Yes<br>(2) No
A13	Have the CHWs administered medicine to your child themselves?	(1) Yes<br>(2) No
A14	Have the CHWs given you other medicine for the child?	(1) Yes<br>(2) No
A15	Have the CHWs told you when and how to give these medicine to the child?	(1) Yes<br>(2) No
A16	Are you satisfied with the atmosphere of trust between you and the CHWs when they come to you?	(1) Yes<br>(2) No
A17	Are you satisfied with the CHWs' behaviour when they come to you?	(1) Yes<br>(2) No
A18	You satisfied with the availability of medicine?	(1) Yes<br>(2) No

A19	Are you satisfied with the care provided to your child?	(1) Yes<br>(2) No
A20	Are there some aspects that you want to make remarks about?	(1) Yes<br>(2) No
