# Supplementary material for: Analysis of the quality of seasonal malaria chemoprevention provided by community health Workers in Boulsa health district, Burkina Faso
Source: BMC Health Serv Res. 2019 Jul 10;19:472. doi: 10.1186/s12913-019-4299-3 (PMC6617895; doi:10.1186/s12913-019-4299-3)
Supplement: Supplementary file 4 — Questionnaire_Socio-demographic characteristics of CHWs. (PDF 120 kb) [file 12913_2019_4299_MOESM4_ESM.pdf]

Questionnaire No. ……

Questionnaire_Socio-demographic characteristics of CHWs

B01	Date	
B02	District	
B03	health centre	
B04	Village	
B05	Names of the CHW	______________________________
B06	Inquirer's name	_________

SOCIO-DEMOGRAPHIC CHARACTERISTICS

B07	How old were you on your last birthday?	/______/_______/
B08	Sex	(1) Male<br>(2) Female
B09	What is your marital status?	(1) Married/ in union<br>(2) Single<br>(3) Separated/ divorced<br>(4) Widower/ widow<br>(5) Other (to specify)/__________
B10	Where do you live?	(1) In this village<br>(2) Another village
B11	What is your schooling level?	(1) Unschooled<br>(2) Literate(local language)<br>(3) Primary school<br>(4) Secondary school<br>(5) University
B12	What is your main occupation?	(1) Farmer<br>(2) Stockbreeder/ poultry raiser<br>(3) Trader<br>(4) Student<br>(5) Other (to specify)
B13	Have you ever worked as an CHW?	(1) Yes<br>(2) No (if *no*, Skip to question B15)
B14	How many years of experience do you have as an CHW?	/________________________
B15	How many SMC programmes have you already partaken as an CD?	/________________________
B16	Have you received any training in SMC this year?	(1) Yes<br>(2) No
